# Size controllable redispersion of sintered Au nanoparticles by using iodohydrocarbon and its implications†
†Electronic supplementary information (ESI) available: Experimental details, XRD patterns, TEM and HRTEM images, XRF. See DOI: 10.1039/c5sc04283f


**DOI:** 10.1039/c5sc04283f

**Published:** 2016-01-27

**Authors:** Xinping Duan, Xuelin Tian, Jinhuo Ke, Yan Yin, Jianwei Zheng, Jin Chen, Zhenming Cao, Zhaoxiong Xie, Youzhu Yuan

**Affiliations:** a State Key Laboratory of Physical Chemistry of Solid Surfaces , National Engineering Laboratory for Green Chemical Production of Alcohols–Ethers–Esters , Collaborative Innovation Center of Chemistry for Energy Materials , College of Chemistry and Chemical Engineering , Xiamen University , Xiamen 361005 , China . Email: yzyuan@xmu.edu.cn

## Abstract

Downsizing large Au particles into small particles with controllable size remains challenging. In this study, we redispersed large sintered Au particles on activated carbon (Au/C) to highly dispersed nanoparticles with uniform distribution and controllable size after treatment with iodohydrocarbons. The Au/C catalyst was conducted for a number of deactivation/regeneration cycles with negligible deterioration in catalytic performance for acetylene hydrochlorination. The redispersion behavior reveals a reverse agglomeration process in the presence of iodohydrocarbons under mild conditions. This behavior is significantly related to the C–I bond dissociation energy (BDE) and adsorption of iodic species on Au particles. A novel protocol for controlling the size and predicting the redispersion efficiency of Au particles is established by correlating with the C–I BDEs of iodohydrocarbons. The molecular-level interpretation of redispersion provides a thorough mechanism based on experimental results. This study presents an efficient method for the easy regeneration of sintered Au-based catalysts for practical applications.

## Abstract 2

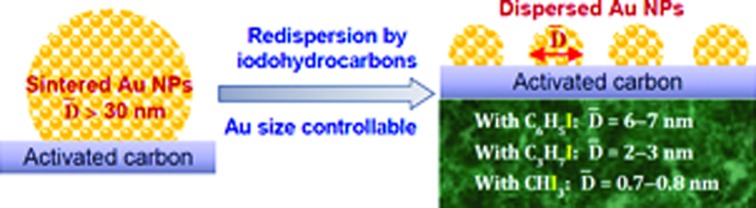
A reverse agglomeration of sintered large Au particles into small ones with size controlled Au nanoparticles has been successfully developed.

## Introduction

Highly dispersed Au particles exhibit outstanding catalytic performance in energy conversion, environmental protection, chemical production, and other fields.^1^ However, Au catalysts without any promoter or functional protection are readily sintered and/or agglomerated, thereby resulting in severe deactivation.^2^ Two mechanistic models, namely, coalescence of small particles and Ostwald ripening for the growth of larger particles at the expense of small particles, have been proposed for Au sintering.^3^ Given that sintering is a common feature, a number of studies have investigated the potential redispersion pathways of large sintered Au particles into small and catalytically active Au nanoparticles (NPs).^3^ Previous studies show that sintered transition and noble metal catalysts can be regenerated through on-line or off-line treatments in the presence of CO, NO, iodomethane (CH_3_I), or ionic liquids.^3^–^7^ For instance, during Au catalyzed methanol carbonylation and ethanol dehydrogenation, large Au particles with an average diameter of 30 nm were dispersed and formed stable Au dimers and trimers in the presence of CH_3_I.^2^,^8^ Sá *et al.*^2^ investigated the dispersion of Au supported on carbon (Au/C) and graphite through CH_3_I treatments at atmospheric pressure and temperature between 50 °C and 240 °C. Goguet *et al.*^5^ demonstrated that sintered Au/C (Au particles with 12 nm to 28 nm in diameter) can be dispersed at the atomic level during carbonylation reaction in the presence of CH_3_I. Au NP redispersion is also achieved under considerably milder conditions and using a mixture of CH_3_X/inert gas feed (X = Br or I).^8^ The proposed reaction mechanism indicates that Au can be oxidized through interaction with halogens. These findings demonstrate the marked transformation of large Au particles into small particles. Nevertheless, the size controlled redispersion of Au particles associated with a deeper understanding the mechanism is necessary and meaningful. Moreover, the straightforward visible redispersion and formation of colloids or nanoclusters from sintered Au particles are sporadic, although directly observed Ostwald ripening or aggregation of metal NPs have been reported by means of *in situ* high-resolution transmission electron microscopy (HRTEM).^9^–^11^

On the other hand, hydrochlorination of acetylene is an important industrial process for synthesizing the vinyl chloride monomer (VCM).^12^ At present, the catalyst that is used for acetylene hydrochlorination consisted of mercuric chloride supported on activated carbon (HgCl_2_/C).^13^,^14^ However, the active component of toxic HgCl_2_ causes serious environmental problems and causes damage to human health as a result of loss of volatile active components under reaction conditions. Hutchings *et al.* reported that monometallic AuCl_3_/C catalyst could present optimal catalytic activity^13^,^15^–^19^ and the initial catalytic activity is higher than that of the HgCl_2_/C and other metal chlorides. However, the stability of monometallic composed AuCl_3_ remains inadequate for long-term practical operation. The deactivation mechanism of Au-based catalysts in acetylene hydrochlorination has demonstrated the sintering of active Au species at temperature levels exceeding 120 °C because of the extraordinary potential reduction of Au^3+^ to Au^0^ during the reaction.^15^–^19^ Methods for regenerating the Au/C catalyst, including employing strong oxidizing process with detrimental agents, such as NO/Cl_2_,^15^ HCl,^13^ and aqua regia^19^ under harsh conditions, has been extensively studied. Rapid and facile regeneration of sintered Au/C catalyst is one of the most highly demanded processes for industrial application. Very recently, Hutchings *et al.* revealed excellent catalytic performance and obtained significant insights for active site and possible reaction mechanism of Au/C-based catalysts with ultralow Au content for acetylene hydrochlorination reaction.^20^ More importantly, they have conducted successful pilot plant evaluation of functional protected Au/C-based catalyst in China. Their full scale reactor containing 790 tubes was loaded with approximately 1.6 t of catalyst and was brought on line and operated under equivalent conditions for more than 4500 h online.^20^

This work focuses on redispersing sintered Au particles with controllable size by using iodohydrocarbons (Fig. S1†). The specific procedure for redispersing a sintered Au/C catalyst was monitored by using time-dependent X-ray diffraction (XRD) patterns and TEM images. The correlation between the C–I bond dissociation energy (BDE) of iodohydrocarbon and the relative redispersion rate was successfully established for the first time. A catalytic test for acetylene hydrochlorination was conducted to demonstrate the high efficiency of the redispersion process. A plausible redispersion mechanism is discussed in this paper.

## Results and discussion

### Redispersion and characterization

**Fig. 1:**
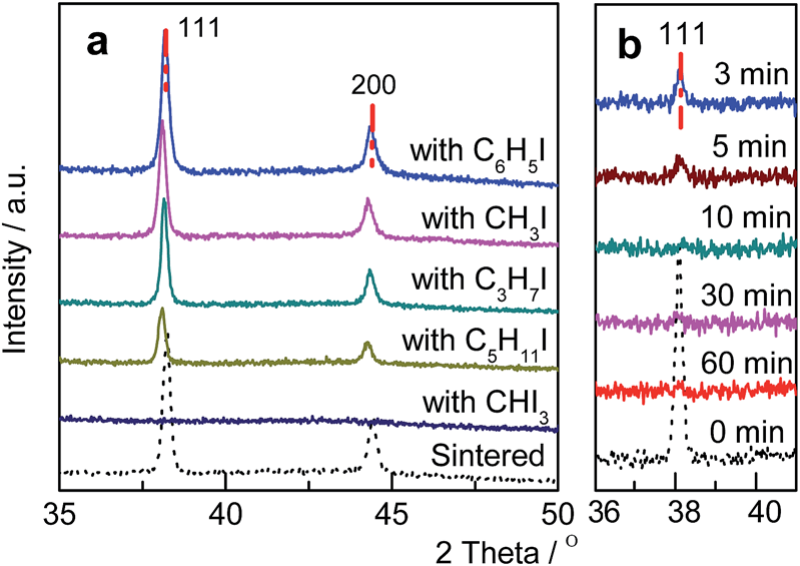
(a) XRD patterns of sintered Au/C catalyst and after treatment with various iodohydrocarbons 40 °C for 3 h; (b) XRD patterns of sintered Au/C catalyst before and after treatment with CHI_3_ at 40 °C as a function of time.

**Fig. 2:**
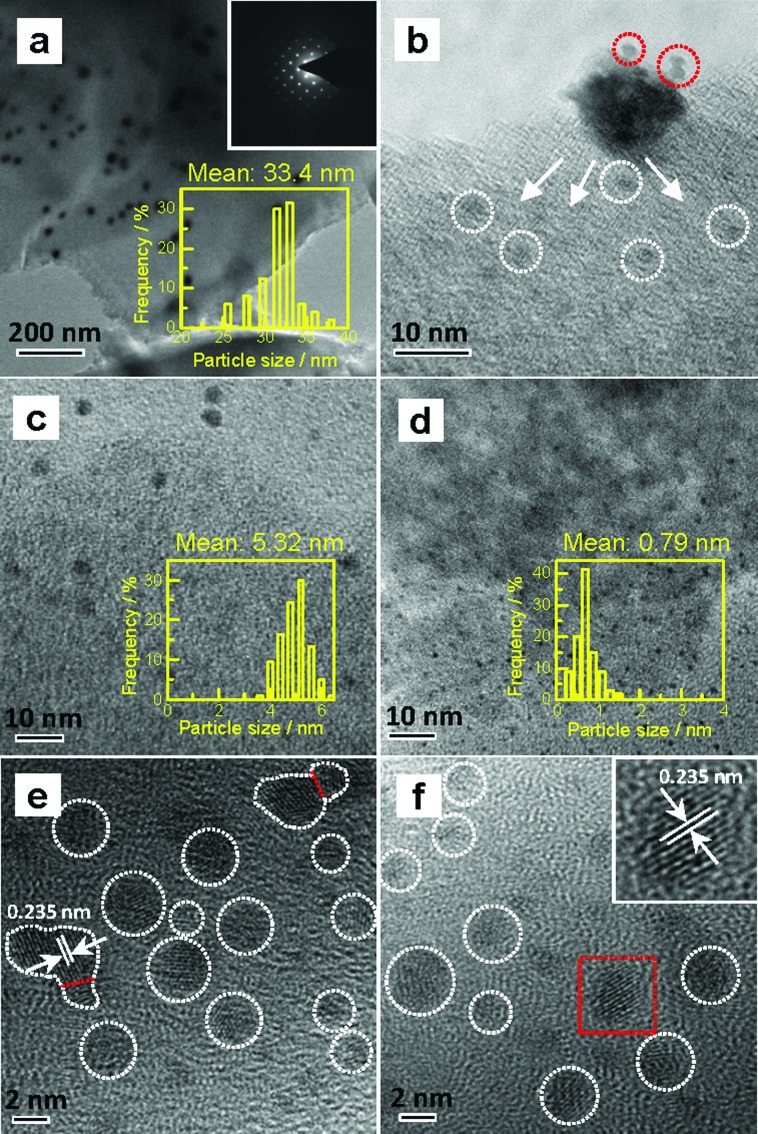
Typical TEM and HRTEM images of randomly selected areas in Au/C catalysts. (a) Sintered catalyst, and the inset shows the selected area electron diffraction (SAED) pattern; (b–d) sintered catalyst after treatment with CHI_3_ at 40 °C for 1, 5, and 60 min, respectively; (e) and (f) HRTEM images of sintered Au/C after treatment with CHI_3_ at 40 °C for 10 and 30 min, respectively; the inset shows the selected area (red rectangle) that represents the lattice spacing of the Au (111) plane.

Various Au/C catalysts, as-prepared, sintered, and treated with different iodohydrocarbons, were prepared through varying given parameters that could affect redispersion rate. Redispersion behavior was captured as single images, time-dependent images, and XRD patterns. Details of preparation and TEM specifications can be found in ESI.† The average diameters of Au particles in the as-prepared and sintered Au/C catalysts were approximately 1.5 (by TEM in Fig. S2†) and 33 nm (by XRD and TEM in Fig. 1a, 2a and S3†), respectively. XRD patterns also revealed the diffraction lines of the Au (111), (200), (220), and (311) lattice planes and a monocrystal feature. When the sintered Au/C catalyst was treated with iodopentane (C_5_H_11_I), iodobenzene (C_6_H_5_I), CH_3_I, and iodopropane (C_3_H_7_I) at 40 °C for 3 h, the intensities of Au diffraction lines decreased according to the type of iodohydrocarbons (Fig. 1a). However, when the sintered Au/C catalyst was treated with triiodomethane (CHI_3_) at 40 °C for 10 min, the diffraction lines of Au NPs almost disappeared because of particle size at less than 3 nm (Fig. 1b). Notably, CHI_3_ is commonly used as a wound disinfectant. Temperature also significantly influenced redispersion, as evidenced by the full width at half maximum (FWHM) and average size of Au NPs (Fig. S5†). Representative time-dependent TEM images and corresponding particle size distribution of Au NPs obtained with CHI_3_ treatment at 40 °C as a function of time are shown in Fig. 2. In terms of treatment temperature, higher temperature levels resulted in the formation of smaller Au NPs.^8^ Furthermore, prolonging the treatment time caused adequate redispersion (Fig. S4, S6, and Table S2†). The influence of oxygen species was studied by addition of *tert*-butylhydroperoxide (TBHP) into the iodohydrocarbon solution to redisperse sintered Au/C. Results show that the presence of active oxygen species minimally affected the redispersion process (Fig. S7†). As an extension, the effects of support on the redispersion behaviors of sintered Au catalysts in liquid phase deserve attention. Therefore, we investigated the aforementioned CHI_3_ treatment strategy to redisperse the sintered Au NPs on carbon materials such as XC-72 and carbon nanotubes (CNTs), as well as oxide supports such as SiO_2_ and CeO_2_ in the liquid phase. The samples obtained were characterized by XRD. In the case of XC-72- and CNTs-supported Au catalysts, the Au particles were transformed from tens of nanometer scales to several nanometers or clusters. By contrast, although a slight decrease was observed in the size of Au particles on CeO_2_ and SiO_2_ supports after the CHI_3_ treatment, the size remained in the submicrometer range. Interestingly, Sá *et al.* observed the redispersion behavior and revealed the applicability of CH_3_I treatments for the dispersion of Au particles on oxide supports in gas phase under higher temperature.^21^

They suggested that the redispersion was strongly influenced by the property of oxide support. The Al_2_O_3_-supported Au catalysts showed a significant decrease in Au particle size, while the SiO_2_-supported Au catalysts downsized insignificantly after the CH_3_I treatment.^21^

To disclose the course of dispersion, we shortened the contact time of Au NPs with CHI_3_ to 1 min and 5 min. Particles with approximately 2 nm in the periphery of larger Au NPs were formed, as shown by the TEM image when the sintered catalyst was treated with CHI_3_ for 1 min (Fig. 2b). The TEM images also indicate that the average particle size of sintered Au/C substantially decreased from approximately 33 nm to 5 nm after treatment of the sintered catalyst with CHI_3_ for 5 min (Fig. 2a and c). Average particle size further decreased to approximately 0.8 nm after treatment for 60 min (Fig. 2d). The typical dark-field scanning TEM images confirmed the transformation of Au NPs (Fig. S6†).

**Fig. 3:**
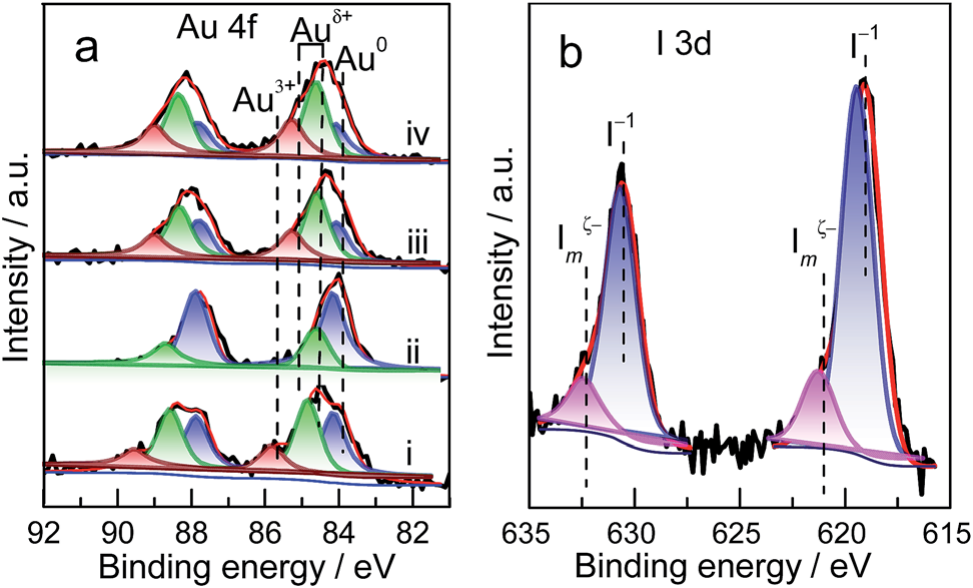
(a) Au 4f XPS spectra of Au/C catalysts after treatment under different conditions: (i) as-prepared; (ii) sintered; (iii) sintered Au/C treated with CHI_3_ at 40 °C for 30 min; and (iv) redispersed Au/C (sample (iii)) activated by HCl gas at 150 °C for 30 min. (b) I 3d XPS spectra of the redispersed Au/C catalyst (sample (iii)).

The preliminary results reveal that Au NP redispersion underwent dissociation as a function of time. A monolayer of iodic species is possibly formed spontaneously on the surface of Au NPs because of the high potential energy of small particles (Fig. 2e and f).^8^,^22^ The adsorption of iodic species on Au NPs decreased the surface potential and subsequently the dissociated iodic species (most probably iodic radicals) from iodohydrocarbons reacted with Au NPs on the carbon support and induced the redispersion process.^3^ Given that Au donates electrons to iodic species, large Au NPs possibly underwent fusion and fragmentation on the support by forming Au–I species. XPS data confirm this hypothesis. In our work, to gain detailed information on the different species and oxidation states of Au (Au^0^, Au^+^, and Au^3+^), the deconvolution and fitting of the Au 4f peak were carefully performed using XPS Peak software. After subtraction of a Shirley background, the peaks were fitted using a nonlinear, least squares routine with mixed Gauss–Lorentz functions. A minimum set of Gauss–Lorentz functions was chosen to obtain a reasonable fit. The quantification and identification of Au species over Au/C catalysts before and after redispersion from XPS are shown in Fig. 3 and Table 1. All evaluated catalysts exhibit Au^0^ and Au^*δ*+^ (0 < *δ* < 3) species, whereas the Au^3+^ species is only present in as-prepared catalyst, and the highest amount of Au^0^ species exists in the sintered Au/C catalyst. Notably, in general, the presence and variation of Au^*δ*+^ in the fresh, sintered, or redispersed Au/C catalyst is attributed to the combination of preparation/redispersion method/processes, dispersion, and incorporation of Au^*δ*+^ and iodine anion. It is not entirely dependent on the size of Au nanoparticles.^5^,^20^ When sintered Au/C was treated with CHI_3_, the Au^*δ*+^ content increased from 26.9% to 72.3%, showing that the Au^0^ species was oxidized to Au^*δ*+^ (Fig. 3a).^5^ Moreover, the I 3d XPS profile suggests the formation of auric iodides (Au_*x*_I_*y*_), accompanied by the presence of I^–^ and polyiodic species (I_*m*_^*ξ*–^, *m* ≥ 1, 0 ≤ *ξ* ≤ 1) that exhibit the binding energy of 619.1 eV and 620.9 eV (Fig. 3b)^23^,^24^ With increasing treatment time, Au content decreased from 0.94 wt% to 0.73 wt%, and gradually became relatively constant at 0.8 wt% (Table S2†).

**Table 1:** Relative chemical composition of all investigated Au catalysts. Elemental concentrations are expressed as atomic percentage

Catalyst	Atomic ratio of Au species/%			Au 4f_7/4_ BE/eV		
	Au^3+^	Au^*δ*+^	Au^0^	Au^3+^	Au^*δ*+^	Au^0^
Fresh	15.5	42.6	41.9	86.1	84.9	83.9
Sintered	0	26.9	73.1	—	84.5	84.1
Redispersed^*a*^	0	72.3	27.7	—	84.4–85.1	83.9
Activated^*b*^	0	74.0	26.0	—	84.4–85.1	84.0

^*a*^Sintered Au/C redispersed with CHI_3_ at 40 °C for 30 min.

^*b*^Redispersed Au/C activated by HCl gas at 150 °C for 30 min.

### Redispersion with size controllability

**Fig. 4:**
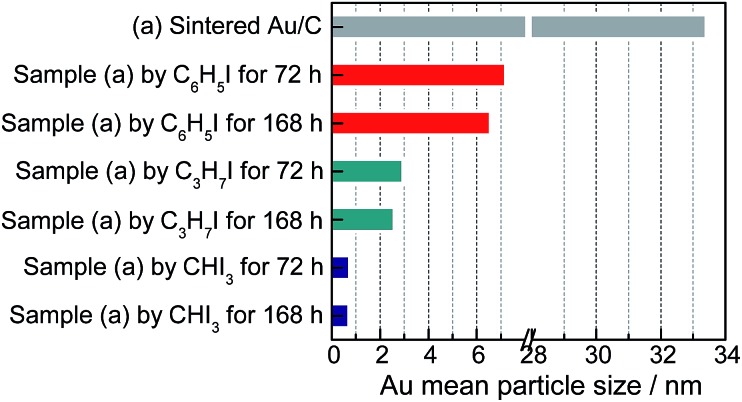
Au average particle size of sintered Au/C before and after redispersion by different iodohydrocarbons for 72 h and 168 h, as indicated by TEM images in Fig. S9.†

The variations in C–I bond strength can be easily considered to be beneficial for redispersing the sintered Au/C catalyst in a controlled manner. Iodohydrocarbons with lower BDE_C–I_ values could produce a higher concentration of iodic species in the liquid phase system,^2^,^25^ and the minimum BDE_C–I_ could induce the formation of ultra-small Au particles. We then redispersed sintered Au/C by using CHI_3_, C_3_H_7_I, and C_6_H_5_I, with gradient decrease in C–I BDEs, at 40 °C for 72 h and 168 h, respectively (Fig. 4). The increase in the FWHM of the long-term treated Au NPs patterns indicates downsizing to smaller nuclei of Au NPs (Fig. S8 and S9†). The FWHM increment and subsequent disappearance of the Bragg reflections were accompanied by the effective redispersion of large Au particles down to the nanocluster level. The HRTEM images reveal that Au particles are highly dispersed and present uniform mean particle sizes of 0.75 nm and 0.72 nm in diameter after CHI_3_ treatment for 72 h and 168 h, respectively (Fig. 2, S9, and S10†). In the cases of C_3_H_7_I and C_6_H_5_I, average sizes of 2.94 nm and 7.21 nm were obtained after treatment for 72 h. These sizes further decreased to 2.58 nm and 6.76 nm after treatment for 168 h, respectively (Fig. S9 and S10†). The difference in size distribution involved different C–I BDEs, which could be attributed to the balance between the adsorption of iodic species and the surface potential energy of Au NPs. Iodohydrocarbons may function as a type of surfactant for size control.^4^,^26^ The long-term redispersion process confirms the formation and controllable size of Au NPs/nanoclusters on the carbon support in a stable state.^2^ Highly uniform dispersion of Au NPs is achieved, suggesting that this protocol benefits the preparation of a highly unified and dispersed catalyst (Fig. 4).

### Correlation of BDEs with redispersion efficiency

**Fig. 5:**
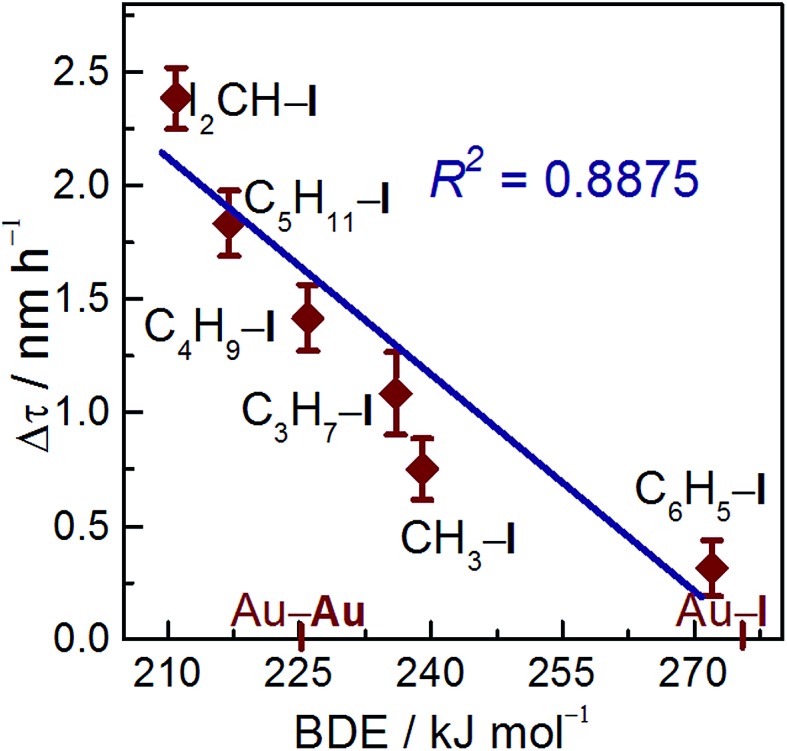
Correlation plots of the dependence of C–I BDEs of iodohydrocarbons on Au NP redispersion rate at 40 °C, derived from variations in radius of Au NPs before and after treatment with iodohydrocarbons.

Redispersion efficiency is based on treatment temperature, time, and nature of the C–I bond energy. To better elucidate the reverse agglomeration behavior, we modeled Au NPs as spherical drops.^27^*R*_S_ and *R*_D_ represent curvature radius of sintered and redispersed Au NPs, respectively (Fig. S1†). Redispersion rate, Δ*τ*, is defined as Δ*τ* = (*R*_S_ – *R*_D_)/Δ*t*, where Δ*t* is the treatment time with iodohydrocarbons. Thus, redispersion rate is related to the treatment temperature and time, as well as to the strength of the C–I bond when the effect of support is excluded.^25^,^28^ The C–I BDE data used in this study were collected from previous experiments, as shown in Table S1.† 29 The BDEs of the iodohydrocarbon used to redisperse the sintered Au/C follows the order, BDE_C_6_H_5_–I_ > BDE_CH_3_–I_ > BDE_C_3_H_7_–I_ > BDE_C_4_H_9_–I_ > BDE_C_5_H_11_–I_ > BDE_CHI_2_–I_. The plots of redispersion rate against C–I BDEs are shown in Fig. 5 (*R*^2^ = 0.8875). The redispersion rate decreased with increasing C–I BDEs of iodohydrocarbons, and the highest redispersion rate was achieved by using CHI_3_, as indicated by minimum BDE_C–I_. In general, iodohydrocarbons with BDE_C–I_ lower than BDE_Au–I_ can theoretically redisperse sintered Au NPs. With increasing difficulty of C–I homolysis in iodohydrocarbons, the concentration of active iodic species (radicals or iodine ions) may decrease.^5^,^30^ As a result of electron donation, Au NPs underwent fusion and fragmentation in the solution.^31^ The spontaneous chemisorption of iodic species on Au NPs is confirmed by electrochemistry results and optical spectra.^22^ The effect of iodohydrocarbons on the dispersion of Au NPs is severely dominated by C–I bond strength, which is strongly correlated with the concentration of active iodic species. Consequently, the results suggest that BDE_C–I_ would function as a descriptor for reverse agglomeration of sintered Au/C. Moreover, the protocol could serve as a potential alternative for preparation of highly uniform Au NP/nanocluster catalysts.

### Mechanism aspect

**Fig. 6:**
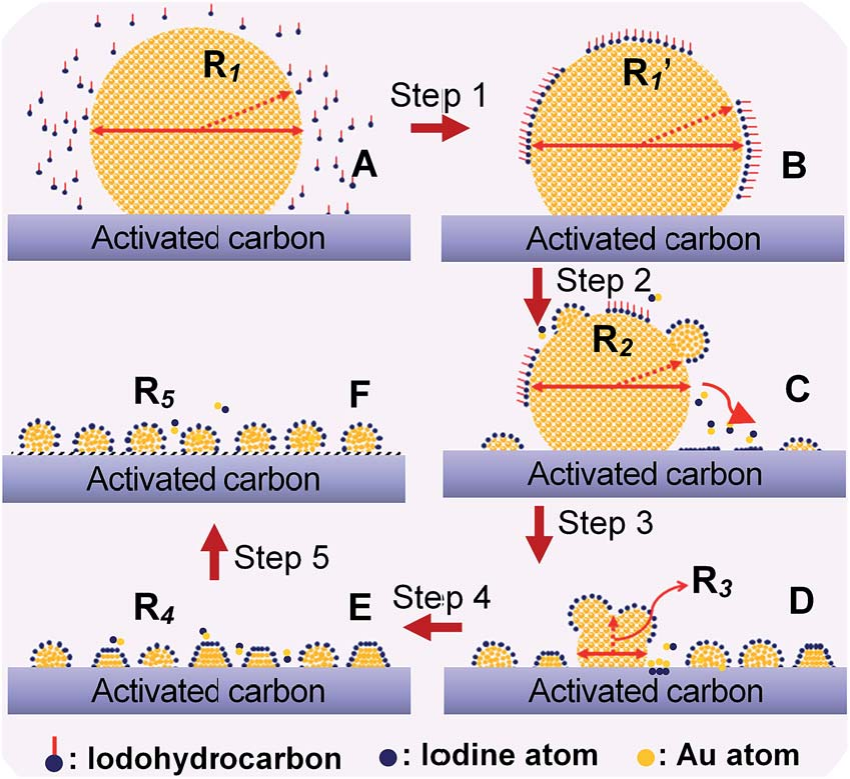
Schematic representation of the fundamental steps underlying adsorption, interaction between iodic species and Au atoms, disintegration, detachment, and changes in size (detachment and redispersion) and/or shape of the Au nanocluster at various stages of redispersion. Sketches C, D, and E represent typical stages during redispersion, as illustrated by TEM images in Fig. 2b, S4 and S9,† respectively.

To demonstrate the general application of the redispersion protocol, we proposed a possible mechanism of Au NP redispersion with size controllability. In general, as an example of CHI_3_ treatment, the fundamental procedures can be interpreted in four steps: chemisorption, disintegration, redispersion, and size control. The rapid chemisorption and interaction of iodohydrocarbons on the surface of large Au NPs initially occurred (Fig. 6, step 1). The bare surfaces of metals tend to readily adsorb adventitious organic materials because adsorbates can decrease the free energy of the interface between metals under ambient conditions.^22^,^32^ The second step involves rapid homolysis dissociation of C–I bond over the Au surface, following slow disintegration and diffusion of main Au particles by forming Au–I species. In the procedure of diffusion, the driving force for Au deposition on carbon surface could be caused by the difference between the reduction potential of intermediate Au_*x*_I_*y*_ and the oxidation potential of rich oxygenated-functional surface of carbon. Therefore, we can rationally deduced that the spontaneous deposition of Au nanoparticles on carbon support during redispersion is due to the redox reaction between carbon and Au_*x*_I_*y*_ intermediates, which is similar to the reaction mechanism between Pt nanoparticles and single-walled carbon nanotubes or graphene oxide.^33^,^34^ The diffusion of Au nanoparticles after nucleation might involve a galvanic-reaction-like process in which the reduction of Au occurs on Au nuclei by the electrons transferred from the carbon surface, with accompanying oxidation of carbon support.^33^ Further disruption was sustained by the ongoing interaction of iodic species with the main Au particles (Fig. 6, steps 2 and 3). Thereafter, equilibrium arises among the typical process of reverse Ostwald ripening of large Au particles, adsorption of Au–I species on activated carbon, and agglomeration of Au–I to form small Au particles (Fig. 6, steps 4 and 5). In this throughout procedure of redispersion, Au–I species assemble for the aggregation energy of Au–Au bond to form a fresh Au NPs/cluster. Moreover, the competition will occur between Au–I species and the Au_*n*_ cluster.^31^,^35^ The final particle size was affected by the complete consumption of large Au NP precursors after long-term redispersion. The final phase involves stabilization of NPs/nanoclusters with size controllable Au NPs. Energetic injection is the physical origin of fusion/fragmentation of Au NPs, which is in accordance with previous studies.^22^,^36^

Overall, the proposed model presents a series of competing factors that affect Au NP dispersion. Among these factors, iodide exhibits the primary mediating function. Iodohydrocarbons (*e.g.*, CHI_3_) may function as a key redispersion segment because the hydrocarbons can strongly and selectively bind to the Au (111) facet and favor the formation of dispersed smaller NPs from the disintegrated complexes. These adsorbates can also alter the interfacial properties with the electrostatic barrier against aggregation and may significantly affect the stability of Au NPs/nanoclusters.^24^,^37^ The chemisorbed iodic radical can neutralize Au NP surface charges and decrease the energy of the iodine-coated NPs.^23^ In addition, considering the electronegativity of Au and iodine (Au 2.4 and I 2.5), Au is considered to exhibit halogen-like behavior in several complexes.^22^,^31^ Through the combination of significant electron affinity of Au (approximately 223 kJ mol^–1^*versus* 295 kJ mol^–1^ of iodine), the Au^+^ ion is readily achievable in the form of Au–I species without need of any oxidants.^31^ This finding is in agreement with the previous results, which showed that TBHP did not affect the redispersion process. In the case of CHI_3_, the relative stability of the characteristic amorphous and ordered clusters was ultimately observed in isolated Au_*n*_ (*n* = 40–200) nanoclusters with a size of 1–2 nm (Fig. S9c and f†).^38^

### Catalytic performance

**Fig. 7:**
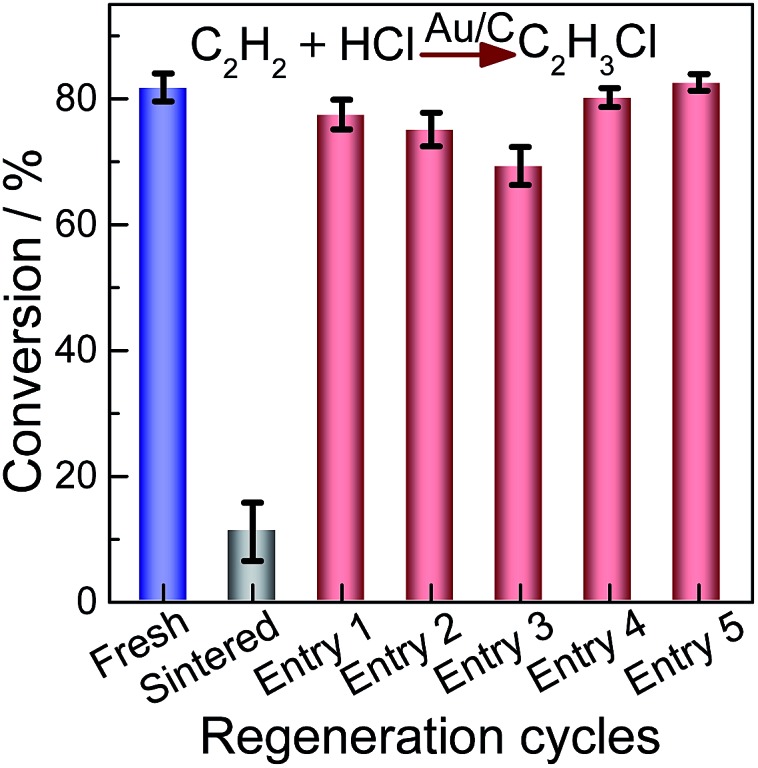
Relative catalytic activity of acetylene hydrochlorination over the fresh, sintered and regenerated Au/C catalysts. Entries 1–3: sintered Au/C regenerated directly with CHI_3_ at 40 °C; Entries 4 and 5: sintered Au/C catalyst regenerated with CHI_3_ containing HAuCl_4_ (0.002 M) at 40 °C.

To further investigate the catalytic activities of the regenerated catalysts, we employ elevated gas hourly space velocity GHSV (C_2_H_2_) compared with that of HgCl_2_ in hydrochlorination. The performance of the redispersed catalyst was evaluated for the hydrochlorination of acetylene as shown in Fig. 7. All data were obtained after 4 h of operation when the steady-state was achieved. The results indicate that acetylene conversion on the as-prepared Au/C catalyst decreased from 81.8% to 11.2% on the sintered sample. The selectivity to VCM was higher than 99% for all examined catalysts. The catalytic activity of sintered Au/C recovered after treatment of CHI_3_ (Fig. 7, entries 1–3), but acetylene conversion was slightly lower than that of the as-prepared catalyst. The decreased conversion could be attributed to the loss of Au stock during redispersion.^12^,^14^ When sintered Au/C is treated with CHI_3_ containing HAuCl_4_ (0.002 M), catalytic activity can be completely restored (Fig. 7, entries 4 and 5). The extended catalysis application was conducted for the durability of redispersed Au/C catalysts. As shown in Fig. S11,† the durability of fresh or regenerated Au/C catalyst was recorded as a function of reaction time as well. All catalysts steadily deactivated with increased reaction time, but no deterioration in catalyst selectivity was observed.^17^,^20^,^39^,^40^ Typical results clearly show that CHI_3_ treatment successfully restores almost full activity to the Au/C catalyst. Although all catalysts evaluated steadily deactivated with increased reaction time, the rate of catalysts regenerated with CHI_3_ is smaller than that of fresh Au/C. The superior durability of regenerated catalysts may be a result of highly dispersed Au NPs (Fig. 1 and 2). Au/C catalysts were successfully operated for a number of deactivation/regeneration cycles with no major deterioration in catalyst performance being observed. In addition, the selectivity of all catalysts evaluated was not affected by the regeneration procedure. Consequently, the Au/C catalysts can be operated for a number of deactivation/regeneration cycles with negligible deterioration in catalytic performance.

## Conclusions

In summary, an easy and rapid protocol for redispersion of sintered Au/C catalysts with Au size controllability is developed. By using of iodohydrocarbons, large Au particles exhibit reverse aggregation behavior that conforms to the reverse Ostwald ripening process. The correlation of the C–I BDEs of iodohydrocarbon with redispersion rate shows that the minimum BDE exhibits optimal redispersion efficiency. The Au/C catalyst can be operated for a number of deactivation/regeneration cycles with negligible deterioration in catalytic performance for acetylene hydrochlorination. These findings would facilitate the redispersion and/or regeneration of Au-based catalysts until the desired particle size is obtained by controlling treatment conditions. The proposed mechanism formulates a molecular-level understanding of critical interfacial events and reveals a new strategy of size-specific Au NP/cluster formation. Our forthcoming study focuses on weakening and elongating the C–I bond to improve dispersion efficiency and size/shape control of Au NPs.

## Supplementary Material

Supplementary information
