# Supplementary material for: Size controllable redispersion of sintered Au nanoparticles by using iodohydrocarbon and its implications
Source: Chem Sci. 2016 Jan 27;7(5):3181–7. doi: 10.1039/c5sc04283f (PMC6005270; doi:10.1039/c5sc04283f)
Supplement: Supplementary file 1 [file SC-007-C5SC04283F-s001.pdf]

Electronic Supplementary Material (ESI) for Chemical Science.
This journal is © The Royal Society of Chemistry 2016

Electronic Supplementary Information (ESI)

Size Controllable Redispersion of Sintered Au Nanoparticles by Using Iodohydrocarbon and Its Implications

Xinping Duan, Xuelin Tian, Jinhuo Ke, Yin Yan, Jianwei zheng, Jin Chen, Zhenming Cao, Zhaoxiong Xie, and Youzhu Yuan*

State Key Laboratory of Physical Chemistry of Solid Surfaces, National Engineering Laboratory for Green Chemical Production of Alcohols-Ethers-Esters, and Collaborative Innovation Center of Chemistry for Energy Materials, College of Chemistry and Chemical Engineering, Xiamen University, Xiamen 361005, China

*Corresponding author. Tel: +86-592-2181659, E-mail: yzyuan@xmu.edu.cn

Materials

Commercially available reagents (such as the metal precursor $HAuCl_4 \cdot 4H_2O$ from Aladdin, assay: 49.7%) were used without purification unless specified. AR-grade iodohydrocarbons, including triiodomethane (CHI_3), iodomethane (CH_3I), *n*-iodopropane (*n*-C_3H_7I), *n*-iodobutane (*n*-C_4H_9I), iodobenzene (C_6H_5I), and *n*-iodopentane (*n*-$C_5H_{11}I$), were obtained from Aladdin and used without further purification. Distilled deionized water, hexane, ethanol, and acetone (AR grade) were used as indicated. Nitrogen, hydrogen chloride, and acetylene (99.999% purity) were purchased from Linde AG.

Catalyst preparation and redispersion procedure

Au/C (1.0 wt%) catalysts were prepared through an incipient wetness impregnation technique as previously described.[1–4] Carbon (40–60 mesh) was initially washed with diluted aqueous HNO_3 (2.0 mol L^{-1}) solution at 80 °C for 5 h to remove Na, Fe, and Cu, which are contaminants for the hydrochlorination reaction. Carbon was filtered and washed with distilled water and dried at 150 °C for 12 h. Requisite amounts of $HAuCl_4 \cdot 4H_2O$ solution in aqua regia (6.3 mL) was added dropwise to the acid-washed carbon (40–60 mesh, 5 g) while stirring. The product was then dried at 110 °C for 12 h and used as a fresh catalyst. Various aging protocols were employed on the supported noble catalysts.[5–8] Selection was conducted to facilitate detection analyses and emphasize the chemical features attributed to sintering of the aged samples. Thus, the physical or chemical properties of the samples are clearly stated when describing the characterization technique used. The fresh catalyst was hydrothermally aged for 5 h at 350 °C in a tubular oven in a flow of dry N_2 (40 mL min^{-1}) with an initial heating ramp of 10 °C min^{-1}. The aged catalysts were then obtained and designated as Au/C. Redispersion treatments of sintered catalysts were carried out in a sealed glass vial. In a typical treatment, 4 mL 30 wt% iodohydrocarbon-acetone solution was first mixed in a sealed sample vial. To it, 200 mg of the sintered catalyst was added to the mixed solution. The samples were vigorously stirred with a magnetic stirrer at a designed temperature under ambient pressure for a fixed time. After treatment, the samples were filtered, washed with hexane, and dried at 110 °C for 12 h. The dried samples were then stored in sealed sample vials.

Catalytic activity evaluation

The catalytic performance of acetylene hydrochlorination was evaluated in a fixed-bed glass microreactor (*i.d.* of 8 mm) under 0.1 MPa.[1,3,9,10] The temperature of the reactor was regulated using a CKW 1100 temperature controller. The reactor was purged with nitrogen for 12 h before the reaction to remove water and air in the system. Hydrogen chloride gas was passed through the reactor at a flow rate of 20 mL min^{-1} to activate the catalyst. After the reactor was heated to 180 °C, (5 mL min^{-1}) hydrogen chloride (5.5 mL min^{-1}) was fed through the heated reactor containing 0.5 mL of the catalyst at a gas hourly space velocity of C_2H_2 (GHSV) of 600 h^{-1}. The reaction products were analyzed using an on-line gas chromatography (GC-2060). Product distribution was determined under the following conditions: chromatographic column, type 2 m × Φ 4 mm; stuffing, GDX-301; column temperature, 120 °C; FID detector; and detector and vaporizer temperature, 150 °C. Given that the individual substance present a high-boiling point and low content, the products of acetylene hydrochlorination were quantified through the peak area normalization method. The conversion of acetylene (X_A) and the selectivity to V_{CM} (S_{VC}) were used as criteria to determine the catalytic performance. As hydrogen chloride was absorbed after the reaction, the volume of the reaction system was set constant during calculation. If the total volume was regarded as a volume unit, X_A and S_{VC} were calculated as follows:

$$X_{\mathrm{A}} = (1 - \varphi_{\mathrm{A1}}) \times 100\% \quad (1)$$

$$S_{\mathrm{VC}} = \varphi_{\mathrm{VC}} / (1 - \varphi_{\mathrm{A1}}) \times 100\% \quad (2)$$

where φ_{A1} is the volume fraction of the remaining acetylene and φ_{VC} is the volume fraction of vinyl chloride. To distinguish catalytic performance, we employed a gas hourly space velocity (GHSV) of C_2H_2 as high as 600 h^{-1}; this velocity is higher than the velocity used for evaluation of conventional $HgCl_2$ catalyst.[11–13]

Catalyst characterization

X-ray diffraction (XRD) patterns of the catalysts were recorded using a PANalytical X'pert Pro Super X-ray diffractometer with Cu K_α radiation (λ = 0.15418 nm) and a scanning angle (2θ) range of 10° to 90°. The tube voltage and current were 40 kV and 30 mA,

respectively. The Au crystallite size was calculated using the Scherrer equation with the full width at half-maximum (FWHM) of the Au (111) diffraction peak at 2θ = 38.4°. Transmission electron microscopy (TEM) images were obtained using a TECNAI F□30 transmission electron microscope operated at an acceleration voltage of 300 kV. The selected area electron diffraction (SAED) pattern was recorded by aligning the electron beam perpendicular to one of the square faces of an individual Au NP. To prepare an appropriate sample for TEM observation, we ultrasonically dispersed the catalyst powder in ethanol at RT for 30 min. The dispersed sample was then transferred onto carbon-coated copper or molybdenum grid through dipping. Particle size distribution was evaluated based on measurements from the full TEM images. The actual gold content of the sample was analyzed through X-ray fluorescence (XRF) spectrometry using a PANalytical AXIOS PW4400 sequential spectrophotometer with an Rh tube as radiation source. Measurements were performed with pressed pellets containing 6 wt.% wax. Characterization was conducted through X-ray photoelectron spectroscopy (XPS) using a Quantum 2000 Scanning ESCA Microprobe instrument (Physical Electronics) equipped with an Al K_α X-ray radiation source ($h\nu$ = 1486.6 eV). The activated catalyst was carefully collected and sealed under Ar atmosphere. The collected sample was compressed into a thin disk in a glove box, and the compressed sample was then transferred to the XPS apparatus analysis chamber. Binding energies were calibrated using the Si 2p peak at 103.7 eV as a reference. Experimental errors were set within ± 0.2 eV.

Bond energy analysis

The most fundamental concepts in organic chemistry include structure, energetics, and reactivity and their interrelationships. The experimental and theoretical values for bond dissociation energies (BDEs) are essential for chemical kinetics, free radical chemistry, organic thermochemistry, and physical organic chemistry. Nevertheless, obtaining reliable data regarding BDEs or strengths of chemical bonds could be complicated and frustrating.[14–16]

The BDE for homolysis of R–X bond is defined as the enthalpy change in the following fission:

$$R–X \rightarrow R + X \quad (3)$$

The BDE, DH^0(R–X), of an R–X bond is derived from the heats of formation of the species involved in the above reaction:

$$DH^0(R–X) = \Delta_f H^0(R) + \Delta_f H^0(X) – \Delta_f H^0(RX) \quad (4)$$

In this case, $\Delta_f H^0$ represents the heats of formation of the respective species in the ideal gas state at standard pressure and reference temperature of 298.15 K. Therefore, BDE is also called the bond dissociation enthalpy. Here, the species R–X represents molecules, radicals, ions, complexes, and clusters. BDEs are commonly referred as bond energies, bond strengths, binding energies, or bond disruption energies (enthalpies). A variety of methods are available for determining BDE values. The experimental BDE values of many important compounds have been measured many times. For instance, the C–H BDE in methane has been determined about 50 times since the 1930s. The BDE values continue to be a source of controversy among scientists. Obtaining data for BDE is only performed experimental studies.[15,16] Although we collected experimental data, including all these information is difficult because most users are concerned with reliable experimental values only.

The present work aims to collect recent experimental data for the given bonds of C–I within iodohydrocarbons, such as CH_3–I, I_2CH–I, and C_3H_7–I. As reliable data for BDEs is difficult to achieve, recent measurements tend to be more generally reliable. The BDEs have been tabulated based on the center atom in the radicals, ions, clusters, and complexes. Table S1 collects the experimental results for BDEs in all evaluated iodohydrocarbons.[14–16]

Table S1 Summary of the C−I BDEs of iodohydrocarbons and iodides.

**The broken bonds (bold character = dissociated atom)**	**BDEs (most recent data; recommended data)**	
**$\Delta_f H^0$(R)/kJ mol^{-1}**	**kJ mol^{-1}**	**references**
CH_3–**I**	238.9 ± 2.1	15
I_2CH–**I**	203.5 ± 33	15
I_3C–**I**	192 ± 50	15
C_2H_5–**I**	233.5 ± 6.3	15
C_3H_7–**I**	236.8 ±4.2	15
C_4H_9–**I**	226.8 ± 4.2	15
C_5H_{11}–**I**	235.7	16
C_6H_5–**I**	272 ±4.2	16
Au–**I**	276	16
Au–**Au**	225.6	16

Table S2 Changes in the Au content as analyzed through XRF and Au NP mean size and full width at half-maximum (FWHM) estimated through XRD/TEM of the sintered Au/C catalyst treated with CHI_3 at 40 °C as a function of time.

**Time / min**	**Au / wt%**	**Time / min**	**Mean size / nm**	**FWHM□**
0	0.94	0	33.4	0.358
60	0.73	3	12.6	0.592
180	0.80	5	8.7	0.639
300	0.81	10	5.4	–
720	0.80	30	1.44	–
1440	0.82	60	0.79	–
10080	0.83	10080	0.72	–

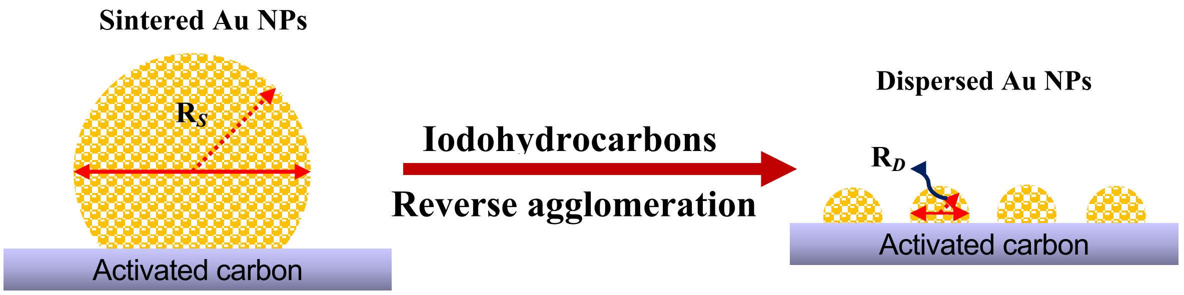

Fig. S1 Schematic of the supported Au NP redispersion in a spherical model with the radius R_S (sintered Au NPs) and R_D (dispersed Au NPs) of curvature. The redispersion rate $\Delta\tau$ is indicated in the following equation:

$$\Delta\tau = (R_S - R_D)/\Delta t \quad (5)$$

where Δt is the treatment time of contact with iodohydrocarbons.

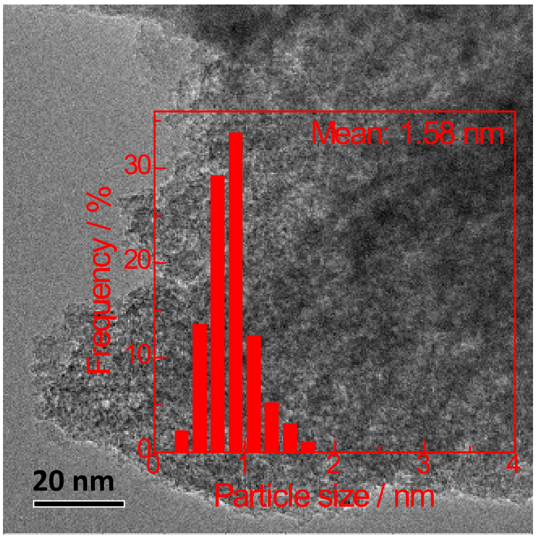

Fig. S2 TEM image and average particle size distribution of as-prepared Au/C catalyst.

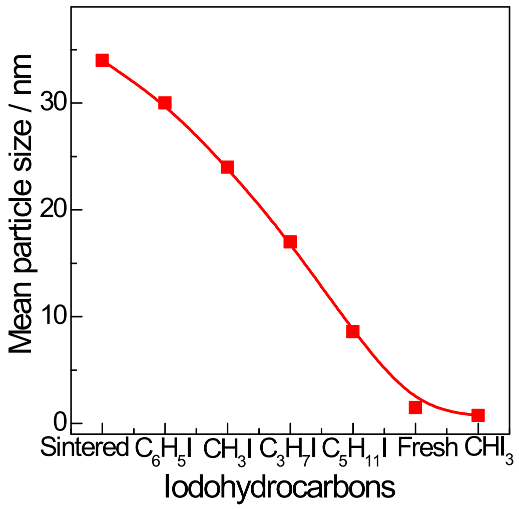

Fig. S3 Average size distribution of Au NPs as indicated in Fig.1a.

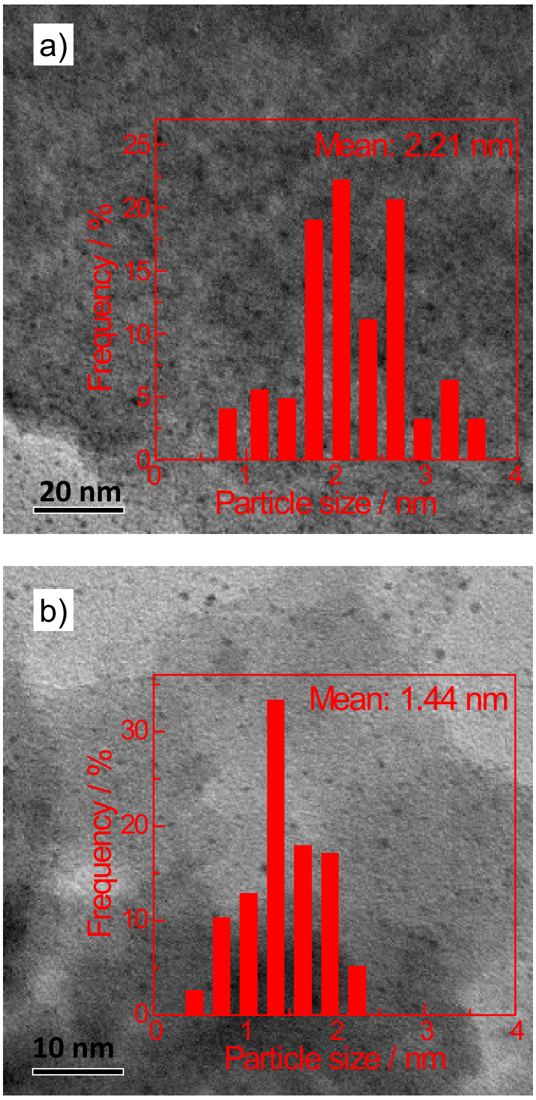

Fig. S4 a) and b) TEM images and corresponding Au particle size distribution of sintered catalyst after treatment with CHI_3 at 40 ºC for 10 min and 30 min, respectively.

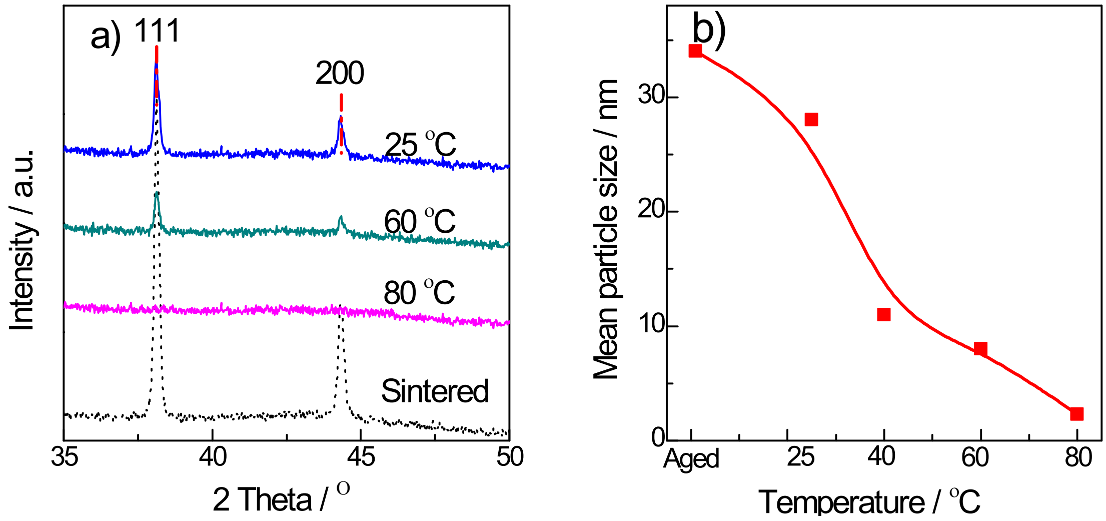

Fig. S5 a) XRD patterns of the Au/C catalysts as a function of temperature in treatment with $C_5H_{11}I$ for 12 h. b) Corresponding Au average particle size of sintered Au/C before and after redispersion indicated in a).

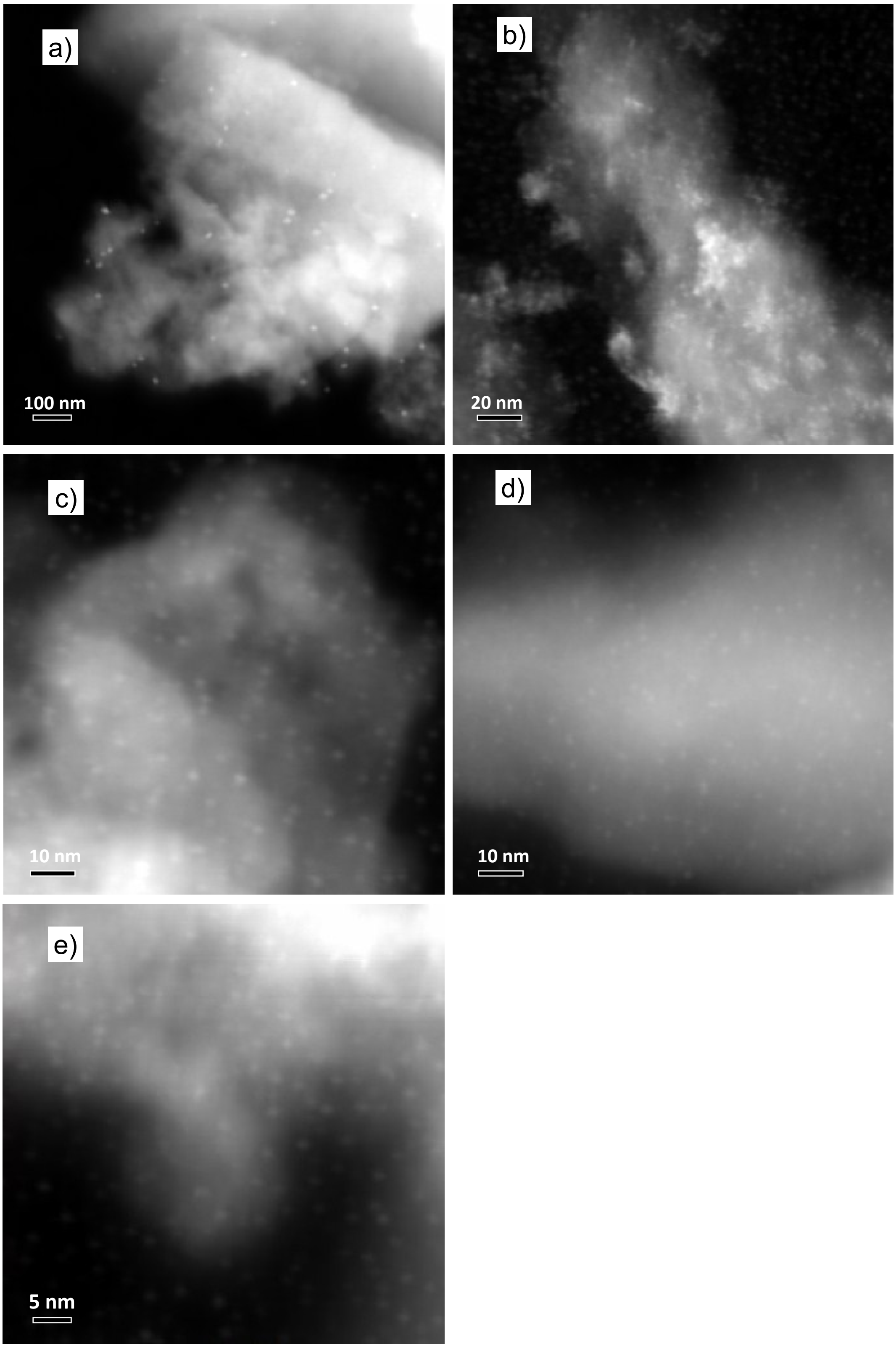

Fig. S6 Typical DF-STEM images of different randomly selected areas of the Au/C catalyst: a) sintered and b−e) sintered catalyst after treatment with CHI_3 at 40 °C for 5, 10, 30, and 60 min, respectively.

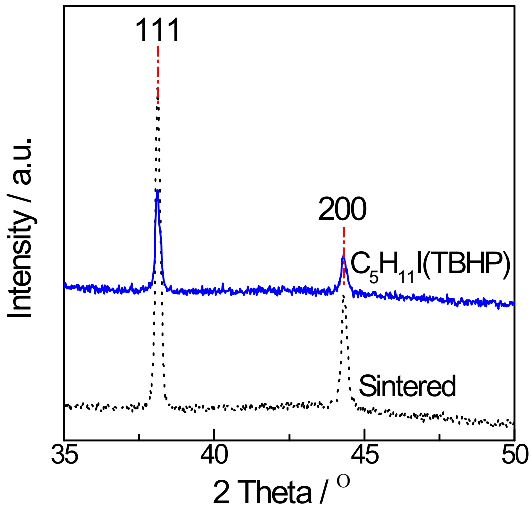

Fig. S7 XRD patterns of the redispered Au/C catalysts after treatment with $C_5H_{11}I$ with addition of t-butylhydroperoxide (TBHP) at 40 °C for 3 h.

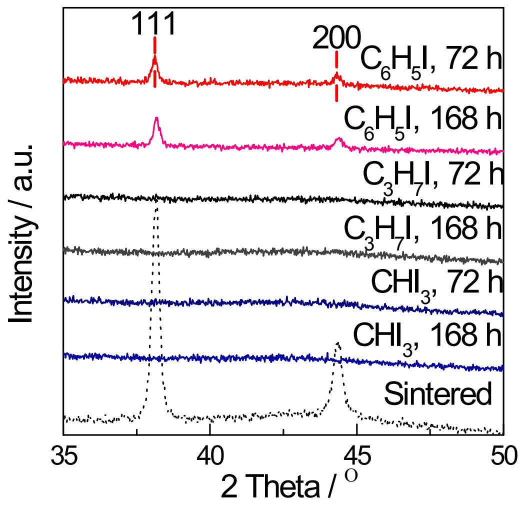

Fig. S8 XRD patterns of the redispered Au/C catalysts after long-term treatment with CHI_3, C_3H_7I and C_6H_5I at 40 °C for 72 h and 168 h, respectively.

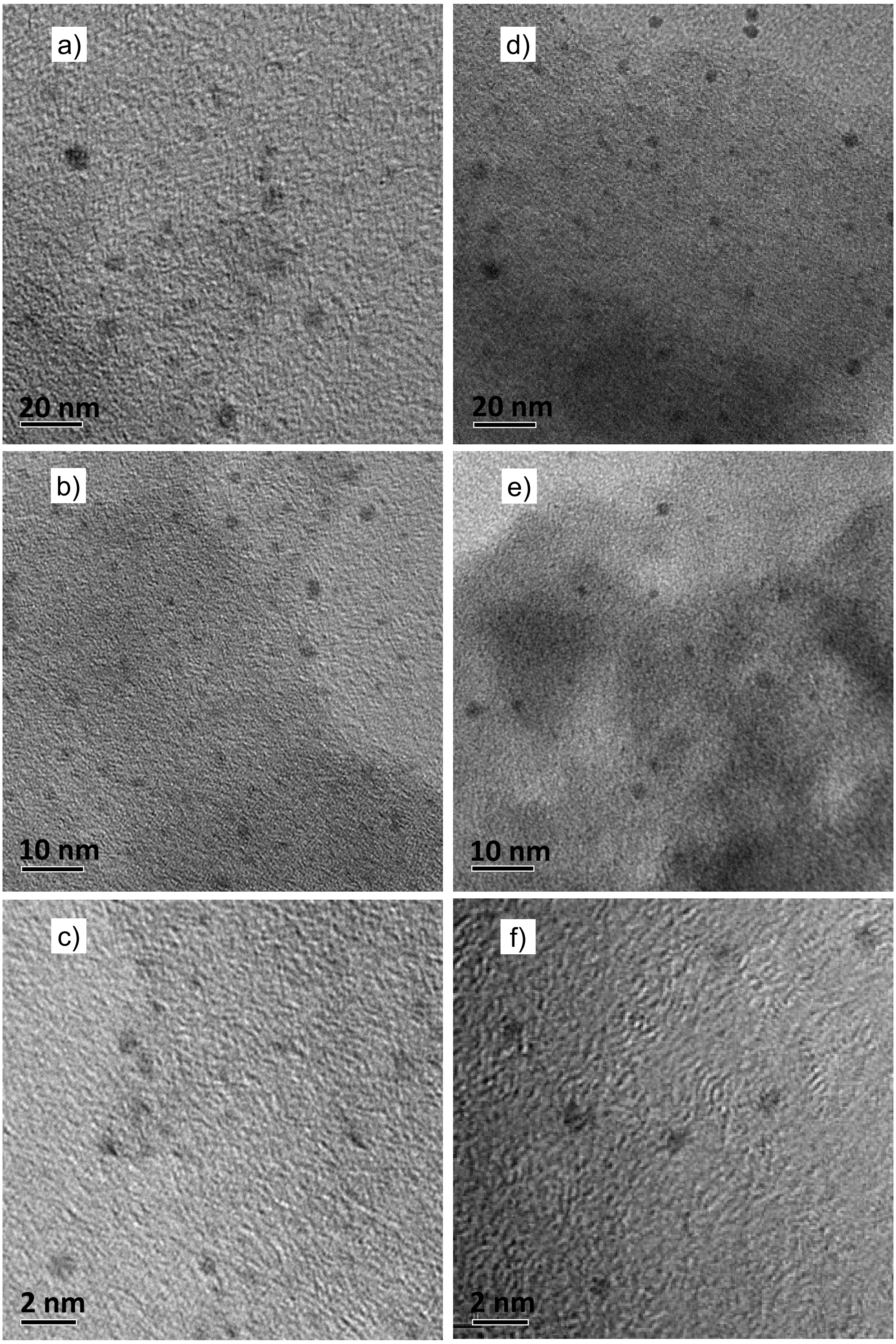

Fig. S9 a–c) and d–f) HRTEM images of sintered Au/C after treatment with C_6H_5I, C_3H_7I, and CHI_3 at 40 °C for 72 and 168 h with their corresponding mean size distributions, respectively.

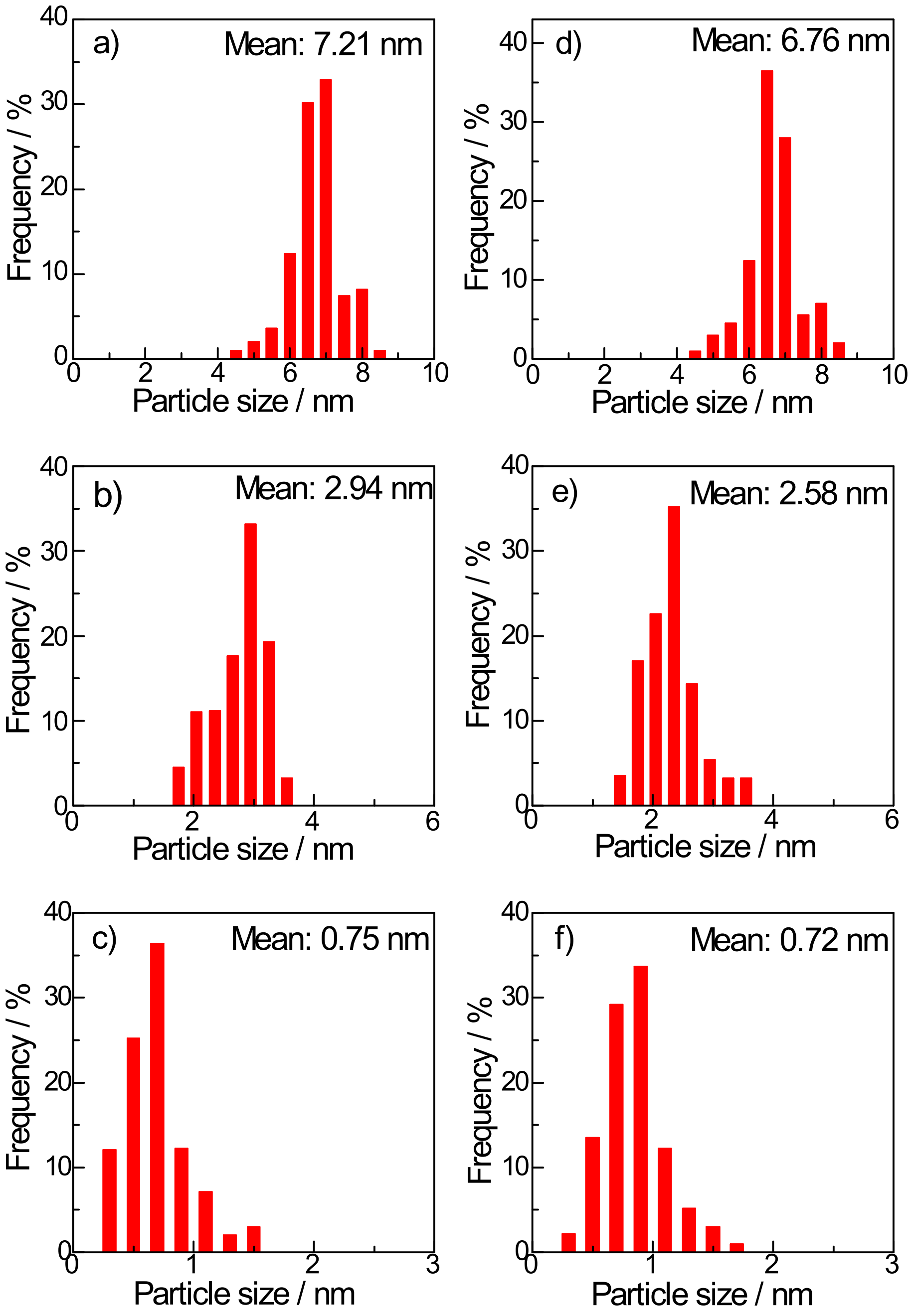

Fig. S10 Corresponding Au particle size distribution histogram in observed TEM images in Fig. S9a–f.

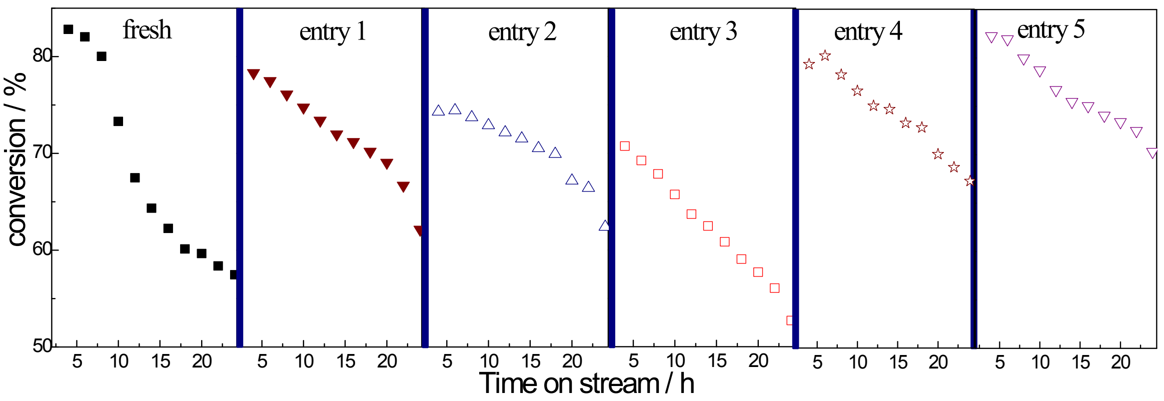

Fig. S11 Durability and activity of acetylene hydrochlorination over the fresh Au/C catalyst and the sintered Au/C catalyst regenerated with CHI_3 treatment for 5 cycles. Reaction conditions: P = 0.1 MPa, T = 180 °C, HCl/C_2H_2= 1.1, GHSV (C_2H_2) = 600 h^{-1}.

References

[1] H. Zhang, B. Dai, X. Wang, W. Li, Y. Han, J. Gu, J. Zhang, *Green Chem.* 2013, **15**, 829.

[2] M. Conte, C. J. Davies, D. J. Morgan, T. E. Davies, D. J. Elias, A. F. Carley, P. Johnston, G. J. Hutchings, *J. Catal.* 2013, **297**, 128.

[3] X. Li, M. Zhu, B. Dai, *Appl. Catal., B* 2013, **142-143**, 234.

[4] M. Conte, A. Carley, G. Attard, A. Herzing, C. Kiely, G. J. Hutchings, *J. Catal.* 2008, **257**, 190.

[5] M. Cargnello, C. Gentilini, T. Montini, E. Fonda, S. Mehraeen, M. Chi, M. Herrera-Collado, N. D. Browning, S. Polizzi, L. Pasquato, P. Fornasiero, *Chem. Mater.* 2010, **22**, 4335.

[6] F. C. Galisteo, R. Mariscal, M. L. Granados, J. L. G. Fierro, R. A. Daley, J. A. Anderson, *Appl. Catal., B.* 2005, **59**, 227.

[7] X. Bokhimi, R. Zanella, A. Morales, V. Maturano, C. Ángeles-Chávez, *J. Phys. Chem. C* 2011, **115**, 5856.

[8] J. A. Anderson, R. A. Daley, S. Y. Christou, A. M. Efstathiou, *Appl. Catal., B* 2006, **64,** 189.

[9] T. V. Krasnyakova, I. V. Zhikharev, R. S. Mitchenko, V. I. Burkhovetski, A. M. Korduban, T. V. Kryshchuk, S. A. Mitchenko, *J. Catal.* 2012, **288**, 33.

[10] H. Zhang, B. Dai, X. Wang, L. Xu, M. Zhu, *J. Ind. Eng. Chem.* 2012, **18**, 49.

[11] K. Chen, L. Kang, M. Zhu, B. Dai, *Catal. Sci. Technol.* 2015, **5**, 1035.

[12] B. Nkosi, N. J. Coville, G. J. Hutchings, *J. Chem. Soc-Chem. Commun.* 1988, 71.

[13] G. J. Hutchings, *J.Catal.* 1985, **96**, 292.

[14] J. A. Kerr, *Chem. Rev.* 1966, **66**, 465.

[15] Y. Luo, *Comprehensive Handbook of Chemical Bond Energies*, CRC Press, Boca Raton, Florida, USA, 2007.

[16] Y. Luo, *Bond Dissociation Energies in Organic Compounds*, CRC Press, Boca Raton, Florida, USA, 2003.
